# Association between blood lead and melanoma: using data from NHANES 2017 to 2023: A cross-sectional study

**DOI:** 10.1097/MD.0000000000047401

**Published:** 2026-01-30

**Authors:** Yanan Tuo, Junchen He, Tao Guo

**Affiliations:** a Department of Dermatology, Tianjin Academy of Traditional Chinese Medicine Affiliated Hospital, Tianjin, China.

**Keywords:** blood lead, melanoma, NHANES

## Abstract

Melanoma ranks as the 4th most common cause of cancer-related mortality globally. To standardize and lower the disease’s incidence, it is critical to continuously investigate and report melanoma risk factors. Lead has been associated with the development of several cancers. Nevertheless, it is still unknown how blood lead levels and the onset of melanoma are related. The purpose of this study was to find out if blood lead and melanoma in US adults were associated. Using information obtained from the 2017 to 2023 National Health and Nutrition Examination Survey, smooth curve fitting and multivariable logistic regression models were used to investigate the association between blood lead and melanoma. The study employed subgroup analysis and interaction testing to examine the stability of this association across populations. Receiver operating characteristic curves were used to evaluate how well blood lead levels and variables predicted the risk of melanoma. In this cross-sectional study, after controlling for all variables, we found a potential association between blood lead levels and melanoma in a study involving 123 melanoma patients. And the risk of developing melanoma increased by 31% for each 1-unit increase in blood lead (1.31 [1.09, 1.56]). The receiver operating characteristic curves showed that model 3’s area under the curve value was 0.618, indicating that the variables may be effective in jointly predicting melanoma prevalence with blood lead levels. According to our research, blood lead exposure is possibly correlated with melanoma in adult US citizens. Further prospective research is required to support the findings of this study.

## 1. Introduction

Melanoma is a potentially fatal cancerous skin growth that starts in the melanocytes. Melanocytes not only influence the skin but also produce pigments in the gastrointestinal tract, eyes, ears, leptomeninges, oral mucous membranes, and genital mucous membranes, as well as mucous membranes in the sinuses.^[1]^ Melanoma is a severe public health concern; a total of 55,500 individuals die from melanoma every year.^[2]^ The incidence rates are on the rise, especially among White people.^[3]^ The age-adjusted incidence of melanoma in the US was 22.7 instances per 100,000 in 2017, and by 2021, it had risen by 5.8%.^[4]^ Although some subtypes of melanoma are unrelated to ultraviolet rays (UV) radiation exposure, exposure to UV radiation from indoor tanning and natural sunlight is usually the primary cause of melanoma.^[5,6]^

Lead is a heavy metal pollutant that can be harmful to human health. Lead comes in both organic and inorganic forms and is not easily broken down. It mostly enters the body through the cutaneous, respiratory, and digestive systems.^[7,8]^ Acute and long-term exposure to lead can have several harmful systemic effects, such as severe anemia, hypertension, type 2 diabetes, lung cancer, urinary incontinence, immune system imbalances, endocrine diseases, delayed skeletal and deciduous dental development, vitamin D deficiency, gastrointestinal problems, and cognitive impairments.^[9–13]^ Lead affects important enzymes, antioxidant reactions, and different hormone functions, all of which have an impact on cell metabolism. Heavy metal-induced oxidative stress responses can also lead to cancer by obstructing deoxyribonucleic acid (DNA) repair.^[13–15]^ Nonetheless, it is still unknown in American cultures whether exposure to lead is associated with melanoma.

The accumulation of the toxic heavy metal lead can have a detrimental impact on human health, even in comparatively modest amounts.^[16]^ Lead can damage a cell’s membrane and structure, but more significantly, it can disrupt DNA transcription.^[17]^ Lead is known to disrupt the functions of several enzymes, including superoxide dismutase, ferrochelatase, catalase, delta-aminolevulinic acid dehydratase, and many more.^[18]^ Lead-induced oxidative stress damages DNA, cell membranes, and cell functioning by increasing the creation of radicals.^[19]^ Lead and skin diseases, including psoriasis and systemic sclerosis, have been reported in previous studies.^[20,21]^ Numerous current studies have shown that environmental variables and skin issues are related.^[22–25]^ As civilization develops and advances, people’s living arrangements and routines also change. Therefore, melanoma risk factors should be actively applied in screening high-risk individuals and regularly reassessed to help reduce the incidence of the disease. Although blood lead is a commonly used biomarker of lead exposure, few studies have examined the association between blood lead and melanoma. To investigate the association between blood lead and melanoma, we conducted a cross-sectional analysis using data from the National Health and Nutrition Examination Survey (NHANES) for the years 2017 to 2023.

## 2. Materials and methods

### 2.1. Study population

**Figure 1.**
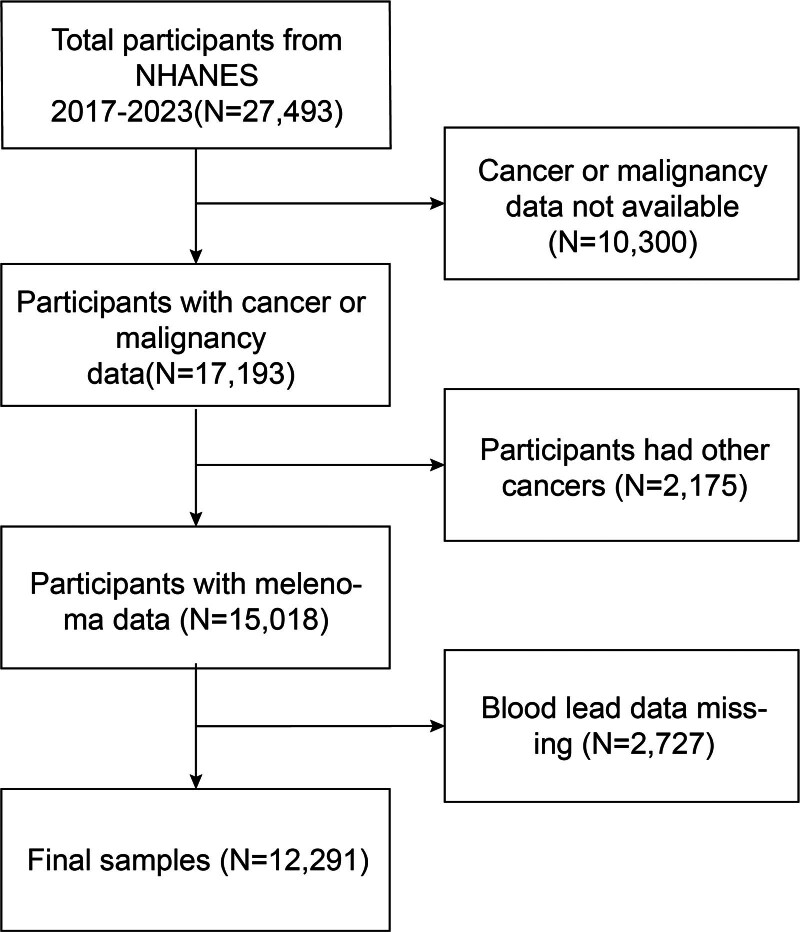
Participants’ flow chart for selection. Participants with missing blood lead or melanoma data were excluded. Additionally, participants with a history of other cancers were also excluded. Ultimately, 12,291 participants completed the survey. NHANES = National Health and Nutrition Examination Survey.

The Centers for Disease Control and Prevention conduct the nationally representative NHANES survey.^[26,27]^ The National Center for Health Statistics Research Ethics Review Board approved the study’s methodology. During the recruitment process, each participant provided written consent.^[28,29]^ For our cross-sectional study, we used NHANES data from the 2017 to 2023 cycles, which correspond to the years when melanoma data were collected. Our study included 27,493 people. We disqualified 2727 people with missing blood lead data and 10,300 people with missing melanoma data. Besides that, 2175 participants who had a history of other cancers were excluded. In the end, 12,291 participants participated in the survey (Fig. 1).

### 2.2. Data on biomarkers

Serum specimens for analysis were received, processed, stored, and shipped by the National Center for Environmental Health, Division of Laboratory Sciences, Centers for Disease Control and Prevention, Atlanta, GA. Venous blood was obtained in order to measure blood lead levels. Prior to measurement, blood samples were kept at −20°C and diluted to a certain quantity. Dilution of the blood in the sample preparation step before analysis is a simple dilution of 1 part sample + 1 part water + 48 parts diluent. In the central laboratory, the blood lead levels were ascertained utilizing a standard procedure and an inductively coupled plasma dynamic reaction cell mass spectrometer (ELAN DRC II, PerkinElmer, Norwalk). The 2 techniques’ higher detection limits for each analyte were applied for both cycles in order to make the combined dataset consistent. Every one of these analytes has 2 variables. The analytic result for the analyte is provided by the other variable prefixed with LBX (for example, LBXBCD). An imputed fill value was entered in the analyte results section for analytes whose analytical results were below the lower limit of detection (for example, LBDBCDLC = 1). This figure is calculated by dividing the square root of 2 by the lower limit of detection.^[2]^ The lead atoms in the droplets from the sample are ionized due to the high energy of the plasma. The NHANES quality assurance and control methods meet the criteria of the 1988 Clinical Laboratory Improvement Act (PBCD_L).

### 2.3. Diagnosis of melanoma

MCQ220 of the Medical Conditions Questionnaire, which asks, “Have you ever been told by a doctor or health professional that you have a malignant tumor?” had participants who answered “Yes” and patients who answered “Melanoma” to MCQ230, “What kind of cancer?” were classified as having melanoma and included in the case group. The control group consisted of participants who answered “No” to question MCQ220.

### 2.4. Covariates

In multivariate models, the associations between blood lead and melanoma might be obscured by other variables. Age, gender, race, education level, income-to-poverty ratio (PIR), drinking alcohol, smoking, diabetes, coronary heart disease, chronic bronchitis, total cholesterol, staying in the shade, using sunscreen, and wearing a long-sleeved shirt were all variables in this research.

### 2.5. Statistical analysis

Continuous variables with a normal distribution were compared between the melanoma and nonmelanoma groups using Student’s *t* tests. The definition of the continuous variables was mean ± standard deviation. The 2 groups were compared using the χ^2^ test for categorical variables that were represented as percentages. Each predictive variable’s melanoma risk was evaluated using univariate analysis. Using logistic regression models, the association between blood lead and melanoma was investigated. A Cook’s distance analysis was performed to assess the robustness of our result. Following the conversion of blood lead from a continuous variable to a categorical variable (quartile), the trend of the association between blood lead and melanoma was examined using a trend test. There were no adjusted variables in model 1; age, gender, and race were adjusted in model 2, and all of the covariates in Table 1 were adjusted in model 3. To investigate the existence of significant interactions between blood lead and melanoma, subgroup analyses were performed based on age, gender, and diabetes. Additionally, stratification factors were taken into consideration as potential moderators of the effect. An interaction term was employed in order to evaluate the heterogeneity using the likelihood ratio test. The results are shown together with the odds ratio (OR), 95% confidence interval (CI), and *P*-value. The nonlinear relationship between blood lead and melanoma was investigated using a smoothing curve fitting. The area under the curve (AUC) values and receiver operating characteristic (ROC) curves were plotted using the “pROC” tool in R (version 1.18.0, PMID:21414208). Statistical significance was defined as a 2-tailed *P*-value < .05. There are no missing values for age, sex, race, education, diabetes status, coronary heart disease, or chronic bronchitis. Poverty to income ratio and total cholesterol had missing values of 13.49%, 1.47%, and 3.34%, respectively, which were interpolated using the mean value. Alcohol drinking, smoking status, staying in shade, wearing a long-sleeved shirt, and using sunscreen had missing values of 12.28%, 2.51%, 35.86%, 36.23%, and 36.23%, respectively, which were interpolated using the median value. For all statistical analyses, we utilized R (https://www.r-project.org) and EmpowerStats (https://www.empowerstats.com).

**Table 1:** Basic characteristics of participants by blood lead among U.S. adults.

Characteristics	Blood lead				*P*-value
	Q1 (N = 3047)	Q2 (N = 3097)	Q3 (N = 3072)	Q4 (N = 3075)	
Age (yr)	39.09 ± 14.32	48.85 ± 16.34	54.77 ± 15.61	59.02 ± 15.02	<.001
Gender (%)					<.001
Male	1016 (33.34%)	1428 (46.11%)	1567 (51.01%)	1784 (58.02%)	
Female	2031 (66.66%)	1669 (53.89%)	1505 (48.99%)	1291 (41.98%)	
Race/ethnicity (%)					<.001
Mexican American	358 (11.75%)	331 (10.69%)	283 (9.21%)	311 (10.11%)	
Other Hispanic	446 (14.64%)	364 (11.75%)	293 (9.54%)	246 (8.00%)	
Non-Hispanic White	1404 (46.08%)	1343 (43.36%)	1301 (42.35%)	1148 (37.33%)	
Non-Hispanic Black	559 (18.35%)	619 (19.99%)	639 (20.80%)	759 (24.68%)	
Other races	280 (9.19%)	440 (14.21%)	556 (18.10%)	611 (19.87%)	
Education level (%)					<.001
< High school	371 (12.17%)	448 (14.46%)	499 (16.25%)	719 (23.38%)	
High school	610 (20.02%)	703 (22.70%)	725 (23.60%)	786 (25.56%)	
> High school	2066 (67.81%)	1946 (62.84%)	1848 (60.15%)	1570 (51.06%)	
Smoked at least 100 cigarettes in life (%)					<.001
Yes	779 (25.57%)	1169 (37.75%)	1381 (44.95%)	1663 (54.08%)	
No	2268 (74.43%)	1928 (62.25%)	1691 (55.05%)	1412 (45.92%)	
Diabetes (%)					<.001
Yes	351 (11.52%)	438 (14.14%)	498 (16.21%)	447 (14.54%)	
No	2696 (88.48%)	2659 (85.86%)	2574 (83.79%)	2628 (85.46%)	
Coronary heart disease (%)					<.001
Yes	49 (1.61%)	112 (3.62%)	147 (4.79%)	187 (6.08%)	
No	2998 (98.39%)	2985 (96.38%)	2925 (95.21%)	2888 (93.92%)	
Family PIR	2.72 ± 1.62	2.84 ± 1.66	2.76 ± 1.66	2.51 ± 1.63	<.001
Total cholesterol (mg/dL)	180.32 ± 37.59	187.20 ± 40.99	189.77 ± 42.77	189.86 ± 42.94	<.001
Had at least 12 alcohol drinks/1 yr (%)					<.001
Yes	81.12	76.35	70.88	62.12	
No	18.88	23.65	29.12	37.88	
Melenoma (%)					.039
Yes	17 (0.56%)	33 (1.07%)	37 (1.20%)	36 (1.17%)	
No	3030 (99.44%)	3064 (98.93%)	3035 (98.80%)	3039 (98.83%)	
Stay in the shade (%)					<.001
Always	217 (8.10%)	138 (6.49%)	151 (8.92%)	137 (9.88%)	
Most of the time	790 (29.49%)	554 (26.06%)	442 (26.12%)	320 (23.09%)	
Sometimes	1166 (43.52%)	950 (44.68%)	702 (41.49%)	572 (41.27%)	
Rarely, or	379 (14.15%)	359 (16.89%)	271 (16.02%)	226 (16.31%)	
Never	127 (4.74%)	125 (5.89%)	126 (7.45%)	131 (9.45%)	
Wear a long-sleeved shirt (%)					<.001
Always	88 (3.30%)	112 (5.30%)	106 (6.29%)	114 (8.28%)	
Most of the time	174 (6.52%)	176 (8.32%)	166 (9.86%)	129 (9.38%)	
Sometimes	660 (24.75%)	543 (25.67%)	482 (28.62%)	405 (29.43%)	
Rarely	804 (30.15%)	557 (26.34%)	411 (24.41%)	307 (22.31%)	
Never	941 (35.29)	727 (34.37%)	519 (30.82%)	421 (30.59%)	
Use sunscreen (%)					<.001
Always	296 (11.10%)	203 (9.60%)	170 (10.10%)	104 (7.56%)	
Most of the time	446 (16.72%)	334 (15.79%)	212 (12.59%)	140 (10.17%)	
Sometimes	679 (25.46%)	455 (21.51%)	332 (19.71%)	250 (18.17%)	
Rarely	519 (19.46%)	408 (19.29%)	297 (17.64%)	203 (14.75%)	
Never	727 (27.26%)	715 (33.81%)	673 (39.96%)	679 (49.35%)	
Chronic bronchitis (%)					<.001
Yes	115 (3.77%)	211 (6.81%)	257 (8.37%)	348 (11.32%)	
No	2932 (96.23%)	2886 (93.19%)	2815 (91.63%)	2727 (88.68%)	

Mean ± SD for continuous variables: the *P*-value was calculated by the weighted linear regression model; (%) for categorical variables: the *P*-value was calculated by the weighted chi-square test.

PIR = income-to-poverty ratio, Q = quartile, SD = standard deviation.

## 3. Results

### 3.1. Baseline characteristics

Of the 27,493 participants, 13,027 were disqualified because of missing or unreliable blood lead and cancer data, and 2175 were excluded because of other cancers, leaving 12,291 for analysis. A total of 123 participants (1.00%) had melanoma, and 12,168 (99.00%) did not have melanoma. Of the 12,291 participants, the mean (standard deviation) age was 50.00 (12.42) years, with 52.85% female and 42.27% non-Hispanic White. Participants with higher levels of blood lead were older (mean age, 59.02 years), were more likely to be male, non-Hispanic Black individuals, and were more likely to have a higher level of education and total cholesterol. In addition, participants with higher levels of blood lead were more likely to have a higher likelihood of diabetes, coronary heart disease, melanoma, smoking, wearing a long-sleeved shirt, and chronic bronchitis. The higher blood lead group may have lower family income, likelihood of staying in the shade, and use of sunscreen (Table 1).

### 3.2. Univariate analysis for melanoma

Table 2 shows the results of the univariate analysis examining factors associated with melanoma. In the univariate analysis, age (OR: 1.08, 95% CI: 1.06–1.09, *P* < .0001), family PIR (OR: 1.31, 95% CI: 1.16–1.47, *P* < .0001), blood lead (OR: 1.23, 95% CI: 1.02–1.45 *P* = .0452) were associated with an increased risk of melanoma. Moreover, those with melanoma also had increased risk factors for coronary heart disease, chronic bronchitis, using sunscreen, wearing a long-sleeved shirt, and alcohol consumption. However, there was no indication that total cholesterol, gender, race, diabetes, or smoking was associated with melanoma risk.

**Table 2:** Univariate analysis for melanoma.

Covariate	Statistics	OR (95% CI)	*P*-value
Age (yr)	50.45 ± 17.06	1.08 (1.06, 1.09)	<.0001
Blood lead (μg/dL)	1.10 ± 1.24	1.23 (1.02, 1.45)	.0452
Total cholesterol (mg/dL)	186.79 ± 41.30	1.00 (0.99, 1.00)	.5700
Gender			.2043
Male (%)	47.15	Reference	
Female (%)	52.85	0.79 (0.56, 1.13)	
Race			.6832
Mexican American	10.44%	Reference	
Other Hispanic	10.98	0.76 (0.20, 2.84)	
Non-Hispanic White	42.27	5.17 (2.10, 12.71)	
Non-Hispanic Black	20.96	0.20 (0.04, 1.02)	
Other race-including multi-racial	15.35	1.22 (0.41, 3.66)	
Education			.0193
<9th grade (%)	6.71	Reference	
9–11th grade (includes 12th grade with no diploma) (%)	9.86	1.59 (0.41, 6.17)	
High school grad/GED or equivalent (%)	22.98	1.66 (0.49, 5.68)	
Some college or AA degree (%)	31.71	3.20 (0.99, 10.32)	
College graduate or above (%)	28.75	4.03 (1.25, 12.93)	
Family PIR	2.71 ± 1.65	1.31 (1.16, 1.47)	<.0001
Smoke at least 100 cigarettes in life			.7112
Yes (%)	40.62	Reference	
No (%)	59.38	0.93 (0.65, 1.34)	
Diabetes			.0912
Yes (%)	14.11	Reference	
No (%)	95.89	0.68 (0.43, 1.06)	
Had at least 12 alcoholic drinks/1 yr			.0190
Yes (%)	73.94	Reference	
No (%)	26.06	0.65 (0.39, 1.06)	
Coronary heart disease			.0065
Yes (%)	4.03	Reference	
No (%)	95.97	0.42 (0.22, 0.78)	
Chronic bronchitis		Reference	.0101
Yes (%)	7.57		
No (%)	92.43	0.51 (0.30, 0.85)	
Use sunscreen (%)			.0015
Always	9.86	Reference	
Most of the time	14.44	0.51 (0.18, 1.47)	
Sometimes	21.88	0.34 (0.12, 0.97)	
Rarely	18.20	0.13 (0.03, 0.63)	
Never	35.63	0.03 (0.00, 0.27)	
Wear a long-sleeved shirt (%)			.03 (.00, .27)
Always	5.36	Reference	
Most of the time	8.22	3.27 (0.38, 28.12)	
Sometimes	26.65	0.80 (0.09, 7.21)	
Rarely	26.51	0.61 (0.06, 5.84)	
Never	33.26	0.03 (0.00, 0.27)	
Stay in the shade (%)			.8174
Always	8.16	Reference	
Most of the time	26.72	3.37 (0.43, 26.16)	
Sometimes	43.00	1.33 (0.16, 10.82)	
Rarely, or	15.67	1.56 (0.16, 15.06)	
Never	6.46	1.39 (0.09, 22.23)	

CI = confidence interval, OR = odds ratio, PIR = income-to-poverty ratio.

### 3.3. Association between blood lead and melanoma

**Figure 2.**
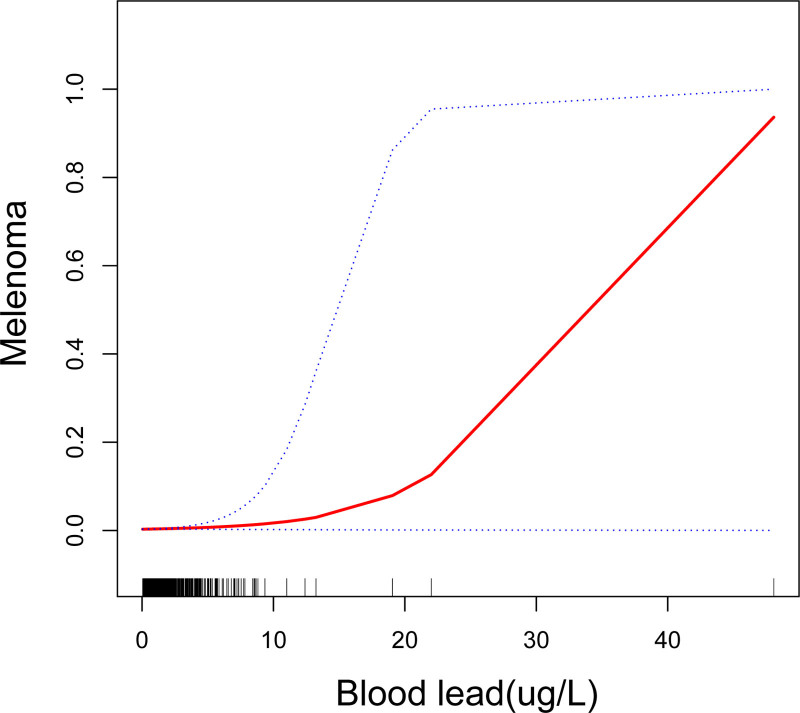
The exposure-response analyses between blood lead and melanoma. The smooth curve fitting results indicate the possibility of an association between melanoma and blood lead levels. The 95% confidence interval and the estimated odds ratio are shown by the upper, lower, and center lines, respectively.

Table 3 displays the correlations between blood lead and melanoma. The unadjusted model showed a possible association between blood lead and melanoma (1.23 [1.02, 1.45]). This correlation is also significant after adjusting for all the covariates in model 3 (1.31 [1.09, 1.56]). After adjusting for all the covariates, the risk of developing melanoma increased by 31% for each 1-unit increase in blood lead. The statistical significance of the aforementioned association remained unaffected by the classification of the blood lead into quartiles (all *P* for trend <.05). The same conclusion was obtained before interpolating the missing values (Table S1, Supplemental Digital Content, https://links.lww.com/MD/R259). The risk of melanoma was 36% higher in those with the highest blood lead quartile (1.36 [1.12, 2.05]) compared to individuals with the lowest. The smooth curve fitting results indicate the possibility of an association between melanoma and blood lead levels (Fig. 2). A Cook’s distance analysis was performed to assess the robustness of our result. As shown in Figure S1, Supplemental Digital Content, https://links.lww.com/MD/R259, the Cook’s distance analysis indicates that all observations exhibit low influence values (maximum Cook’s distance = 0.05), with no single observation demonstrating excessive influence.

**Table 3:** Association between blood lead and melanoma.

Blood lead	Melanoma OR (95% CI)	*P* for trend
Crude model (model 1)		
Continuous	1.23 (1.02, 1.45)	
Categories		.0452
Quartile 1	1 (ref)	
Quartile 2	1.92 (1.07, 3.45)	
Quartile 3	2.17 (1.22, 3.87)	
Quartile 4	2.11 (1.18, 3.77)	
Minimally adjusted model (model 2)		
Continuous	1.56 (1.31, 1.96)	
Categories		.0084
Quartile 1	1 (ref)	
Quartile 2	2.01 (1.12, 3.62)	
Quartile 3	2.31 (1.30, 4.13)	
Quartile 4	2.48 (1.38, 4.43)	
Fully adjusted model (model 3)		
Continuous	1.31 (1.09, 1.56)	
Categories		.0120
Quartile 1	1 (ref)	
Quartile 2	1.25 (1.01, 1.43)	
Quartile 3	1.29 (1.10, 1.97)	
Quartile 4	1.36 (1.12, 2.05)	

Model 1: no covariates were adjusted.

Model 2: age, gender, and race were adjusted.

Model 3: age, gender, race, education level, PIR, drinking alcohol, smoking, diabetes, coronary heart disease, chronic bronchitis, total cholesterol, staying in the shade, wearing a long-sleeved shirt, and using sunscreen were adjusted.

CI = confidence interval, OR = odds ratio, PIR = income-to-poverty ratio, Q = quartile.

### 3.4. Subgroup analyses

To find out if the association between blood lead and melanoma was consistent in the overall population and to identify any possible differences in demographic contexts, we conducted subgroup analysis and interaction tests stratified by gender, age, and diabetes (Table 4). According to our findings, there was no significant difference in the association between melanoma and blood lead between subgroups under Bonferroni correction (α = 0.05, *m* = 18)(*P* > α/*m*).

**Table 4:** Subgroup analysis of the association between blood lead and melanoma.

Subgroup	Melanoma (OR [95% CI])	*P* for interaction	Bonferroni
Sex		.5878	0.0028
Male	1.71 (0.60, 4.87), 0.3134		
Female	1.06 (0.24, 4.75), 0.9351		
Age		.2995	
<60 yr	1.01 (0.76, 1.35)		
≥60 yr	0.78 (0.60, 1.01)		
Diabetes		.9654	
Yes	1.00 (0.71, 1.41), 0.8857		
No	1.20 (0.63, 2.45), 0.5413		

Age, gender, race, education level, PIR, drinking alcohol, smoking, diabetes, coronary heart disease, chronic bronchitis, total cholesterol, staying in the shade, wearing a long-sleeved shirt, and using sunscreen were adjusted.

CI = confidence interval, OR = odds ratio.

### 3.5. ROC curves

**Figure 3.**
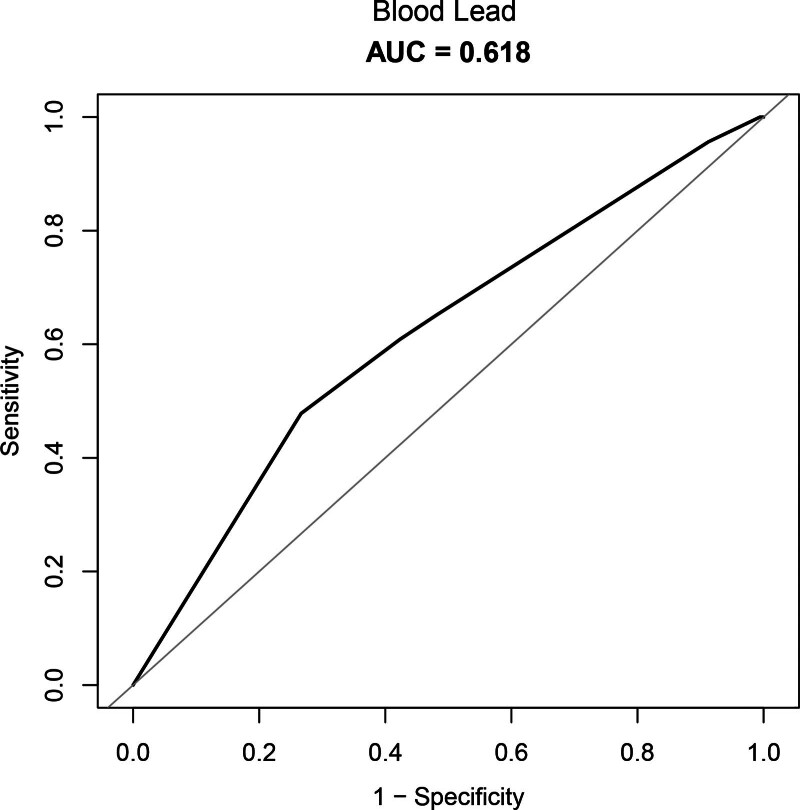
ROC curve of model 3. Model 3’s AUC values were 0.618. Blood lead levels may have predictive value for melanoma occurrence when combined with other variables. AUC = area under the curve, ROC = receiver operating characteristic.

The ROC curve is regarded as the most crucial instrument for assessing how well predictive models and medical diagnostic tests function. The test or model’s predictive power increases with the AUC value’s proximity to 1. An AUC value of 0.511 was observed using only lead content as a variable (Figure S2, Supplemental Digital Content, https://links.lww.com/MD/R259). Model 3’s AUC values were 0.618 (Fig. 3). The findings showed that the occurrence of melanoma could be better predicted by blood lead levels in conjunction with other variables.

## 4. Discussion

The cross-sectional investigation comprising 12,291 representative adults revealed an association between blood lead and melanoma, suggesting that elevated blood lead levels could raise the risk of melanoma development. Model fitting and summary analysis were performed using information on blood lead levels, melanoma, and a few other variables. The connection between each clinical trait and the risk of melanoma was evaluated using the chi-square (categorical variables) and rank-sum (continuous variables) tests. Research findings indicate that age, blood lead levels, educational attainment, alcohol consumption habits, PIR index, coronary heart disease, chronic bronchitis, wearing long-sleeved shirts, sunscreen usage frequency, and arthritis may all be associated with the occurrence of melanoma (*P* < .05). The association between blood lead levels and the risk of melanoma was then evaluated using 3 fitted models. The OR values in all the models were >1, and the *P*-values for blood lead levels were <.05. According to our research, blood lead may be used clinically to diagnose the severity and risk of melanoma.

To our knowledge, only a limited number of studies have examined the association between lead exposure and melanoma incidence. Lead is a common heavy metal. There are several methods for it to enter and build up in the human body. It can result in neuropsychiatric disorders as well as the advancement of malignancy.^[30]^ When blood lead levels in adults and children reach 5 μg/dL, adverse health effects are identified.^[16]^ Our research indicates that blood lead levels may be associated with melanoma in the general population. The chance of getting melanoma rose by 56% for every unit rise in blood lead, even after controlling for partial confounding variables. Lead and its compounds are classified by the International Agency for Research on Cancer as “probable” human carcinogens (group 2A).^[31]^ An animal study demonstrated that in animals injected with a single dose of melanoma cells containing 10 µmol/L lead chloride, tumor growth rates were significantly slower than in the control group. Following a single injection of lead chloride and sodium selenite (concentration > 10 µmol/L), both tumor weight and volume were significantly lower than in the control group.^[32]^ However, in a prospective cohort of women with nonoccupational exposure, there were 239 incident cancers diagnosed following an average follow-up of 6 years. Compared to women with the lowest blood lead concentration, women with greater blood lead levels had a significantly increased risk of developing any cancer (hazard ratio = 1.46; 95% CI: 1.006–2.13; *P* = .046).^[33]^ Based on data from human studies, the patient group’s serum levels of lead and chromium tended to be higher but did not reach statistical significance. Serum lead concentrations in basal cell carcinoma patients were found to be substantially higher than those in the control group in the examination of heavy metal levels in the various forms of skin cancer.^[34]^ White et al recently showed a greater risk of postmenopausal breast cancer in women exposed to increasing levels of lead, which is consistent with the earlier findings.^[35]^ A study examining the relationship between urinary lead and cancer mortality revealed that lead levels in the urine were predictive of death from cancer in relation to lead carcinogenesis.^[36]^ Furthermore, Awadalla et al examined the lead levels in 132 controls and 268 bladder cancer patients. It was discovered that bladder cancer patients had statistically significantly higher blood lead concentrations.^[37]^

The fundamental processes by which exposure to heavy metals promotes cancer are yet unclear. Numerous studies have shown that lead exposure can impact T and B lymphocyte development as well as natural killer cells, which play a critical anticancer function.^[38]^ Lead also has an impact on the activity of multifunctional serine/threonine protein kinase C, which is crucial for controlling a number of cellular functions such as proliferation, apoptosis, and exocytosis.^[7]^ In addition to causing direct harm, lead lowers mitochondrial potential, raises calcium ion and reactive oxygen species levels in cells, and inhibits apoptosis by releasing cytochrome.^[39]^

The strength of this study is the large number of participants from 2 consecutive NHANES cycles. One of the most important aspects of the study is the use of a complex multi-stage probability sampling strategy, which enhances the study’s reliability and representativeness. There are also certain limitations. Reliance on self-reported melanoma conditions might result in reporting bias and a lack of clinical confirmation, even while a medical professional or health care practitioner makes the diagnosis, but validation through medical records or histopathology data is not feasible. Second, the cross-sectional design makes it impossible to prove causation. Reverse causation states that therapy or lifestyle modifications (e.g., drug use or dietary changes) may result in raised blood lead levels in melanoma patients. Thirdly, it’s possible that blood lead levels don’t accurately represent the body’s total lead accumulation. Since melanoma has a long latency, current blood lead levels may not represent relevant past exposure. This conceptual mismatch weakens the biological plausibility of the association. Fourth, the study design may potentially lead to residual confounding even if we took into account a large number of potential confounders. The NHANES database does not contain data on UV indices, occupational exposures, family history, and genetic factors related to place of residence. All of this data may have an impact on the results. A large number of samples are missing, which may lead to reduced statistical validity and biased results. However, the results from all the models were comparable.

## 5. Conclusions

According to our investigation, there is possibly an association between blood lead and melanoma in adult Americans. Our study might provide insight into possible preventative and therapeutic approaches for melanoma. Our conclusions on this research challenge need to be supported by further excellent prospective studies.

## Acknowledgments

We express our gratitude to all participants in the study.

## Author contributions

**Funding acquisition:** Junchen He.

**Methodology:** Tao Guo.

**Software:** Junchen He.

**Project administration:** Yanan Tuo, Tao Guo.

**Resources:** Yanan Tuo, Tao Guo.

**Validation:** Yanan Tuo, Tao Guo.

**Visualization:** Yanan Tuo, Junchen He.

**Investigation:** Junchen He, Tao Guo.

**Writing – original draft:** Yanan Tuo.

## Supplementary Material


