# Supplementary material for: Association between blood lead and melanoma: using data from NHANES 2017 to 2023: A cross-sectional study
Source: Medicine (Baltimore). 2026 Jan 30;105(5):e47401. doi: 10.1097/MD.0000000000047401 (PMC12863770; doi:10.1097/MD.0000000000047401)
Supplement: Supplementary file 1 [file medi-105-e47401-s001.docx]

**Supplemental Table 1:** Association between blood lead and melanoma.

| **Blood lead** | **melanoma** | **P for trend** |
| --- | --- | --- |
|  | **OR (95% CI)** |  |
| Crude model (Model 1)  Continuous | 1.67 (1.15, 1.85) |  |
| Categories  Quartile 1  Quartile 2  Quartile 3  Quartile 4  Minimally adjusted model (Model 2)  Continuous  Categories  Quartile 1  Quartile 2  Quartile 3  Quartile 4  Fully adjusted model (Model 3)  Continuous  Categories  Quartile 1  Quartile 2  Quartile 3  Quartile 4 | 1 (ref)  1.90 (1.21, 3.05)  2.21(1.32, 3.92)  2.51 (1.48,3.29)  1.56 (1.31, 1.96)  1 (ref)  1.61 (1.02, 3.53)  1.92 (1.07, 3.45)  2.18 (1.40, 3.66)  1.45 (1.15, 1.73)  1 (ref)  1.27 (1.15, 1.76)  1.34 (1.10, 1.87)  1.79 (1.02,2.79) | 0.0327  0.0152  0.0215 |

Model 1: no covariates were adjusted. Model 2: age, gender, and race were adjusted. Model 3:age, gender, race, education level, PIR, drinking alcohol, smoking, diabetes, coronary heart disease, chronic bronchitis, total cholesterol, staying in the shade, [wearing a long-sleeved shirt](E:/黑素瘤/OA_Table%202.docx#DEQ034C), and [using sunscreen](E:/黑素瘤/OA_Table%202.docx#DEQ034D) were adjusted. Abbreviation: PIR, the ratio of income to poverty; Q, quartile.

**Supplemental Figure 1. Cook’s distance plot for the logistic regression model of melanoma and blood lead.**

**

**

**Supplemental Figure 2. ROC curve using only blood lead content as a variable. AUC value is 0.511.**

**
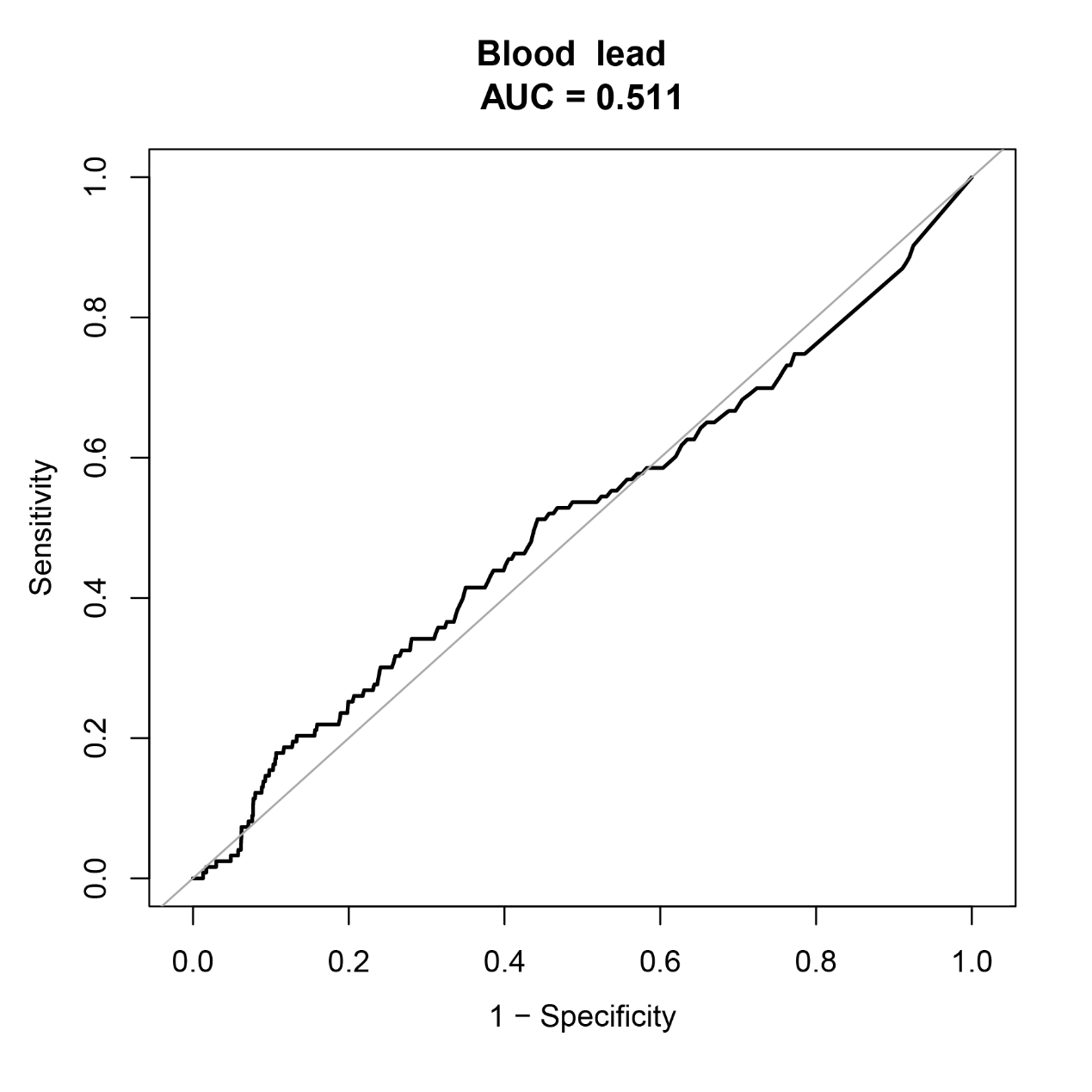
**
